# Methylation of *SERPINA1* gene promoter may predict chronic obstructive pulmonary disease in patients affected by acute coronary syndrome

**DOI:** 10.1186/s13148-021-01066-w

**Published:** 2021-04-15

**Authors:** John Charles Rotondo, Giorgio Aquila, Lucia Oton-Gonzalez, Rita Selvatici, Paola Rizzo, Monica De Mattei, Rita Pavasini, Mauro Tognon, Gianluca Calogero Campo, Fernanda Martini

**Affiliations:** 1 Department of Medical Sciences, University of Ferrara, Ferrara, Italy; 2 Department of Translational Medicine and for Romagna, University of Ferrara, Ferrara, Italy; 3 Cardiology Unit, Azienda Ospedaliera Universitaria Di Ferrara, Ferrara, Italy; 4 Laboratory for Technologies of Advanced Therapies (LTTA), University of Ferrara, 70 Eliporto Street, 44121 Ferrara, Italy

**Keywords:** Chronic obstructive pulmonary disease, Acute coronary syndrome, COPD, ACS, SERPINA1, Alpha 1-antitrypsin

## Abstract

**Background:**

Diagnostic biomarkers for detecting chronic obstructive pulmonary disease (COPD) in acute coronary syndrome (ACS) patients are not available. *SERPINA1*, coding for the most potent circulating anti-inflammatory protein in the lung, has been found to be differentially methylated in blood cells from COPD patients. This study aimed to investigate the methylation profile of *SERPINA1* in blood cells from ACS patients, with (COPD+) or without COPD (COPD−).

**Methods:**

Blood samples were from 115 ACS patients, including 30 COPD+ and 85 COPD− according to lung function phenotype, obtained with spirometry. DNA treated with sodium bisulfite was PCR-amplified at *SERPINA1* promoter region. Methylation analysis was carried out by sequencing the PCR products. Lymphocytes count in ACS patients was recorded at hospital admission and discharge.

**Results:**

*SERPINA1* was hypermethylated in 24/30 (80%) COPD+ and 48/85 (56.5%) COPD− (*p* < 0.05). Interestingly, at hospital discharge, lymphocytes count was higher in COPD− patients carrying *SERPINA1* hypermethylated (1.98 × 10^3^ ± 0.6 cell/µl) than in COPD− carrying *SERPINA1* hypomethylated (1.7 × 10^3^ ± 0.48 cell/µl) (*p* < 0.05).

**Conclusions:**

*SERPINA1* is hypermethylated in blood cells from COPD+ patients. COPD− carrying *SERPINA1* hypermethylated and high lymphocytes count may be at risk of COPD development. Therefore, *SERPINA1* hypermethylation may represent a potential biomarker for predicting COPD development in ACS patients.

## Introduction

Chronic obstructive pulmonary disease (COPD) is a chronic inflammatory lung disease that causes obstructed airflow from the lungs [1]. It is caused by long-term exposure to particulate matter, most often from cigarette smoke [1]. Acute coronary syndrome (ACS) results from acute obstruction of a coronary artery [2]. Several studies suggest that ACS patients with concomitant COPD have a poor prognosis [3–5]. However, COPD is frequently undiagnosed in ACS patients, with a significant delay in the treatment and a negative impact on short- and long-term prognosis [5]. The gold standard for COPD diagnosis is the spirometry.

*SERPINA1* gene encodes for Alpha-1 antitrypsin (AAT), a blood protease of 52 kDa constitutively released from the hepatocytes [6]. AAT is involved in inflammatory processes [7, 8]. Indeed, this protein plays a protective role on the healthy cells adjacent to the inflamed tissue where it inhibits different proteases, including the elastase produced by neutrophils [7]. The activity of AAT is very high in the lower respiratory tract where it provides more than 90% of the defenses against the elastolytic load of neutrophils [9, 10]. The relevance of AAT in COPD is evident in individuals carrying mutations in *SERPINA1* gene where absence or alteration of the protein in association with cigarette consumption predisposes to the risk of developing COPD [11].

*SERPINA1* gene is 12.2 kb and maps on chromosome 14q32.1 [12]. It consists of 7 exons called IA, IB, IC and II–III–IV–V, and six introns [13]. Transcriptional regulation occurs in exons IA, IB and IC, in a tissue-specific manner [13]. IA and IB regulate transcription in the monocytes and macrophages and IC in hepatocytes [14, 15]. *SERPINA1* is also an inducible gene upon activating its inflammation-responsive promoter in hepatocytes, monocytes and macrophages [7, 16, 17]. High levels of circulating AAT, fourfold to sixfold higher than baseline levels, are present during the course of inflammation, infections and late pregnancy [18–21].

In a recent study we have performed a comprehensive assessment of *SERPINA1* gene promoter methylation profile in peripheral blood mononuclear cells (PBMCs) from healthy subjects [21]. We showed that *SERPINA1* gene promoter is hypermethylated in healthy individuals, such as blood donors, but hypomethylated in pregnant women at the third trimester of pregnancy. These findings suggest that *SERPINA1* gene is normally silenced in blood cells of healthy individuals, but it is induced under emergency conditions, such as late pregnancy, when women are highly exposed to the risk of inflammations and infections [21].

Based on our recent findings, in this study we aimed to verify whether methylation of *SERPINA1* gene promoter may differ between ACS patients with COPD (COPD+) and without COPD (COPD−). To this aim, the methylation profile of *SERPINA1* gene promoter was investigated in blood cells from COPD+ and COPD− patients.

## Methods

### Sample population

Blood samples were collected from 115 ACS patients (mean age ± standard deviation [SD], 65 ± 9 yrs) at the University Hospital of Ferrara, Ferrara, Italy. The inclusion criteria comprised smokers or former smokers (≥ 10 pack/years) patients hospitalized with a clinical diagnosis of ACS, as defined by current European guidelines [22]. Exclusion criteria included previous diagnosis of COPD and/or asthma, known pulmonary diseases other than COPD, ongoing pneumonia, ongoing heart failure, documented or suspicion of malignant disease, life expectancy < 1 year, recent thoracic trauma.

### Definition of undiagnosed COPD

Clinical and laboratory data as well as blood samples were collected for all patients during hospitalization. Two months after discharge, all patients underwent spirometry to verify the presence of COPD. A spirometry was performed by two expert pulmonologists and revised by an independent reviewer blinded to patients’ clinical conditions and outcomes. Spirometry was performed according to standardized procedures [23]. Briefly, COPD was diagnosed in the presence of: (1) exposure to risk factors for the disease (all patients enrolled in the study were current or former smokers); (2) presence of chronic respiratory symptoms (mainly shortness of breath, cough and sputum) and (3) post-bronchodilator fixed ratio forced expiratory volume at first second (FEV1)/forced vital capacity (FVC) < 0.7.

### Blood collection and leukocyte count

A venous blood sample was collected from all ACS patients at the time of both admission and hospital discharge. Leukocyte count was performed by flow cytometry with the automated cell counter Sysmex XN. When needed, a blood smear was prepared for microscopic evaluation and accurate quantification of total lymphocytes count.

### DNA extraction and DNA PCR suitability

DNA was isolated from total blood using QIAamp DNA Blood Mini Kit (Qiagen, Milan, Italy), as described [24]. After purification, DNA was quantified by spectrophotometric reading (NanoDrop 2000, Thermo Scientific) [21] and then evaluated for its PCR suitability by amplification for *β-globin* gene sequence [25].

### Treatment of DNAs with sodium bisulfite and SERPINA1 PCR

**Fig. 1:**
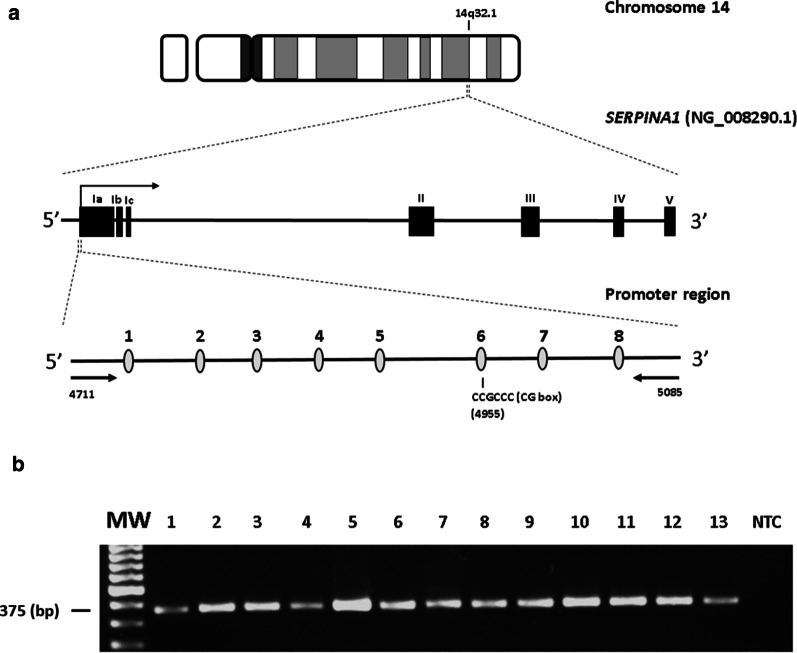
**a** Genomic structure of chromosome 14, *SERPINA1* gene and *SERPINA1* promoter region. Upper line: graphical representation of chromosome 14. *SERPINA1* gene is located on the long arm of the chromosome at 14q32.1. Middle line: graphical representation of *SERPINA1* gene (GenBank accession number NG_008290.1). The gene includes seven exons (Ia-Ic, II, III, IV and V) and six introns. Filled-in boxes and horizontal arrow indicate gene exons and orientation, respectively. Bottom line: analyzed *SERPINA1* promoter sequence with 8 CpGs including the 6th CpG of the amplified region located at the CG-box consensus sequence. The horizontal arrows represent the primers. **b** Bisulfite PCR of *SERPINA1* promoter region. MW, molecular weight; NTC, no template control. Columns 1–13, representative bisulfite-treated DNAs from acute coronary syndrome (ACS) patients with chronic obstructive pulmonary disease (COPD+, columns 1–6) and without COPD (COPD−, columns 7–13)

Methylation analysis was carried out by DNA treatment with sodium bisulfite, using the Epitect Bisulfite kit (Qiagen, Milan, Italy), as reported [21]. Treated DNA was purified with DNA purification columns (Epitect Bisulfite kit, Qiagen, Milan, Italy) and then subjected to PCR amplification for *SERPINA1* gene promoter (GenBank accession number: NG_008290.1). Briefly, 150 ng of DNA was amplified using the primers forward 5′-TTTTGGTTTAGTTTAGGATTTTGAGG-3′ and reverse 5′-ACCTACCAATTATTAATACCAAATCTATAC-3′ [21, 26]. These primers amplify a promoter region of 375 bp (GenBank accession number: NG_008290.1, position 4711–5085), which contains 8 CpG dinucleotides. Among these CpGs, CpG number 1 (CpG-1) and CpG-8 have been previously found hypomethylated in association with lower average lung function phenotypes and COPD [26], while CpG-6 belongs to a CCGCCC-box regulatory consensus sequence of the promoter region (Fig. 1a) [21]. PCR program was: 10 min of denaturation at 95 °C followed by 40 cycles of 1 min at 95 °C, 1 min at 65 °C and 2 min at 72 °C and a final extension for 5 min at 72 °C [21]. A PCR-negative control containing distilled water without DNA, was included per reaction. PCR products were run onto 2% agarose gel electrophoresis and stained with ethidium bromide.

### DNA sequencing of SERPINA1 PCR products

To evaluate the methylation levels of *SERPINA1* promoter, PCR products were sequenced by direct sequencing [27]. Briefly, PCR products were purified using the QIAquick PCR Purification Kit (Qiagen, Milan, Italy) and then subjected to direct sequencing using the automated ABI-Prism-3130X DNA sequencer (Applied Biosystems, Monza, Italy) [25], as performed before [28]. *SERPINA1* sequences showing more than or equal to 5 out of 8 methylated CpGs (≥ 50%) were considered hypermethylated [27, 29].

### Statistical analysis

Continuous data were tested for normal distribution with the Kolmogorov–Smirnov test. Normally distributed values were presented as mean ± SD and compared by *t* test [30]. Otherwise, median [interquartile range] and Mann–Whitney *U* were used. The Chi-square trend test with Yate’s correction [31] was used to compare the observed *SERPINA1* epigenotypes, i.e., DNA hypermethylation and hypomethylation, among COPD+ and COPD− groups. Lymphocyte values were analyzed with D'Agostino-Pearson test for normality, and groups were compared employing the nonparametric Mann–Whitney *U* test [32, 33]. Spearman correlation analysis was applied to evaluate age-related variations in *SERPINA1* promoter methylation by matching the level of methylation to the age of each individual [30]. Multiple linear regression model has been used to estimate the associations between *SERPINA1* epigenotypes and lymphocytes count. Statistical analyses were carried out employing the R software package (version 3.5.0) and using the GraphPad Prism for Windows (version 5.0, GraphPad) [34]. *p* values less than 0.05 (*p* < 0.05) were considered statistically significant [35].

## Results

### ACS patients

Population characteristic of ACS patients stratified according to the presence of COPD are reported in Table 1. Overall, 115 patients were included in the analysis. Of those, after spirometry, 30 (26%) were confirmed with COPD. Of note, only age, respiratory health screening questionnaire (RSHQ), FEV_1_ and pack-year resulted to be significantly different between COPD− (*n* = 85) and COPD+ (*n* = 30): age 63 ± 9 versus 70 ± 8 years, *p* = 0.001; RSHQ > 19 29% versus 57%, *p* = 0.008; FEV_1_ (mean *L* ± SD) 2.9 ± 0.7 versus 2.0 ± 0.5, *p* < 0.001; pack-year, mean ± SD 34 ± 23 vs 45 ± 38, *p* < 0.05 in COPD− vs. COPD+, respectively.

**Table 1:** Population characteristics according to the presence of undiagnosed chronic obstructive pulmonary disease (COPD)

General characteristics, mean ± SD	COPD− (*n* = 85)	COPD+ (*n* = 30)	*p* value
Age, years	63 ± 9	70 ± 8	**0.001**
BMI, Kg/m^2^	27.7 ± 4.2	27 ± 3.6	0.420
Male, n° (%)	73 (86)	23 (77)	0.243
*CV risk factors and comorbidities, n° (%)*			
Diabetes	19 (22)	9 (30)	0.401
Hypertension	58 (68)	18 (60)	0.413
Family history of CAD	23 (27)	8 (27)	0.967
Dyslipidemia	47 (55)	13 (43)	0.260
Pre-MI	10 (12)	8 (27)	0.053
Pre-PCI	17 (20)	3 (10)	0.214
Pre-CABG	3 (4)	2 (7)	0.469
AF	8 (9)	3 (10)	0.925
PAD	4 (5)	2 (7)	0.678
*COPD parameters*			
RHSQ > 19, n° (%)	25 (29)	17 (57)	**0.008**
FEV1, L, mean ± SD	2.9 ± 0.7	2.0 ± 0.5	** < 0.001**
Smoker, n° (%)	39 (46)	15 (50)	0.698
Former smokers, n° (%)	46 (54)	15 (50)	0.698
Pack years, mean ± SD	34 ± 23	45 ± 38	**0.049**
*Clinical presentation and coronary vessel disease, n° (%)*			
STEMI	38 (45)	15 (50)	0.617
NSTEMI	27 (32)	10 (33)	0.874
Unstable angina	20 (24)	5 (17)	0.433
Descending artery	67 (79)	18 (60)	0.051
Circumflex artery	44 (52)	12 (40)	0.268
Right coronary artery	61 (72)	18 (60)	0.232
Left main	12 (14)	5 (17)	0.551
*Therapy, n° (%)*			
Aspirin	84 (99)	30(100)	0.553
P2Y12 inhibitor	85 (100)	30(100)	0.999
Beta-blockers	72 (85)	28(93)	0.228
ACE inhibitors	77 (91)	28(93)	0.646
Statins	83 (98)	28(93)	0.268
*Laboratory data, mean* ± *SD*			
Troponin T, ng/dl	1.99 ± 2.7	2.3 ± 4.3	0.643
CK-MB peak	78 ± 119	90 ± 141	0.676
WBC admission, u/μl	9.4 ± 3.05	9.5 ± 2.94	0.998
WBC discharge, u/μl	8.48 ± 2.21	7.91 ± 1.88	0.209
Lymphocytes, u/μl, admission	2.14 ± 0.99	1.95 ± 0.96	0.365
Lymphocytes, u/μl, discharge	1.85 ± 0.57	1.74 ± 0.73	0.352
Neutrophils, u/μl, admission	6.11 ± 2.66	5.95 ± 2.06	0.763
Neutrophils, u/μl, discharge	5.56 ± 1.84	4.91 ± 1.36	0.081
NLR, admission	3.47 ± 2.17	3.78 ± 2.51	0.516
NLR, discharge	3.24 ± 1.44	3.39 ± 1.88	0.639

COPD+: ACS patients, with chronic obstructive pulmonary disease (COPD). COPD−: ACS patients without COPD. WBC: white blood cell, SD: standard deviation, ACE: angiotensin-converting enzyme, STEMI: ST-elevation myocardial infarction, NSTEMI: non-ST-elevation myocardial infarction, MI: myocardial infarction, PCI: percutaneous coronary intervention, CABG: coronary artery bypass graft, AF: atrial fibrillation, PAD: peripheral artery disease, COPD: chronic obstructive pulmonary disease, FEV1: forced expiratory volume at first second, FVC: forced vital capacity, RHSQ: respiratory health screening questionnaire, HDL: high-density lipoprotein, CAD: coronary artery disease, CV: cardiovascular, BMI: body mass index, BSA: body surface area. u/μl: cell/μl, NLR: neutrophil/lymphocyte ratio

### SERPINA1 gene promoter methylation analysis

In order to profile the methylation status, *SERPINA1* was PCR amplified in a promoter region containing 8 CpG dinucleotides (Fig. 1a). PCR amplifications were efficiently obtained in all blood samples from all patients (Fig. 1b).

**Fig. 2:**
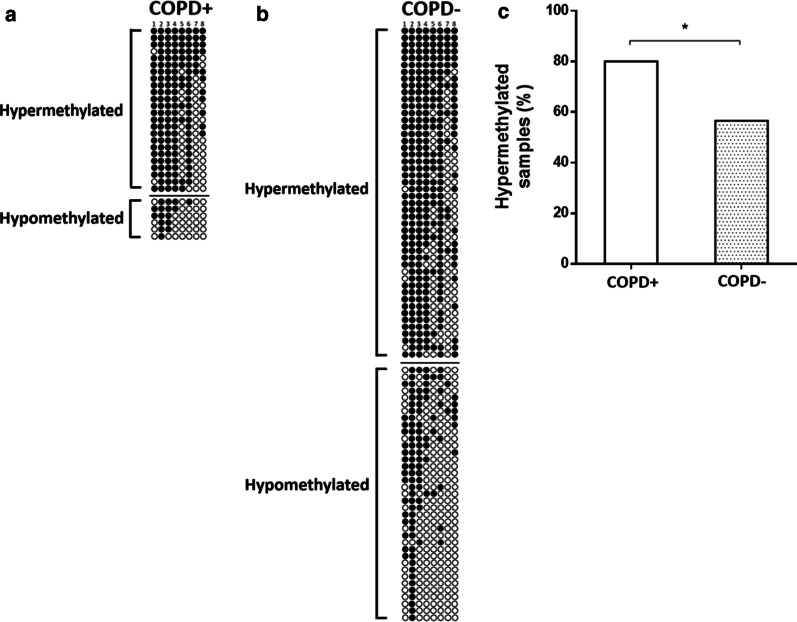
Bisulfite–polymerase chain reaction (PCR) sequencing of the *SERPINA1* promoter of DNAs from blood samples belonging to acute coronary syndrome (ACS) patients **a** with chronic obstructive pulmonary disease (COPD+) and **b** without COPD (COPD−). In both panels: stratification of bisulfite–PCR sequences according to ACS subgroup and *SERPINA1* hyper- and hypomethylation status. Filled-in and clear circles represent methylated and unmethylated CpGs, respectively. The CpGs within the *SERPINA1* promoter are numbered across the top of the grid. Each row represents one PCR product/analyzed sequence. Samples showing more than or equal to 5 out of 8 (≥ 50%) methylated CpGs were considered hypermethylated; **c** frequencies of *SERPINA1* promoter hypermethylation in COPD+ and COPD− samples. *p < 0.05

The prevalence of methylation in *SERPINA1* gene promoter was evaluated by sequencing analysis of PCR products. *SERPINA1* gene promoter was found to be hypermethylated in 72/115 (62.6%) of ACS patients. *SERPINA1* was found to be hypermethylated in 24/30 (80%) COPD+ and in 48/85 (56.5%) COPD− (Fig. 2a, b). The difference in *SERPINA1* hypermethylation between COPD+ and COPD− ACS patients resulted statistically significant (*p* < 0.05; Fig. 2c).

**Fig. 3:**
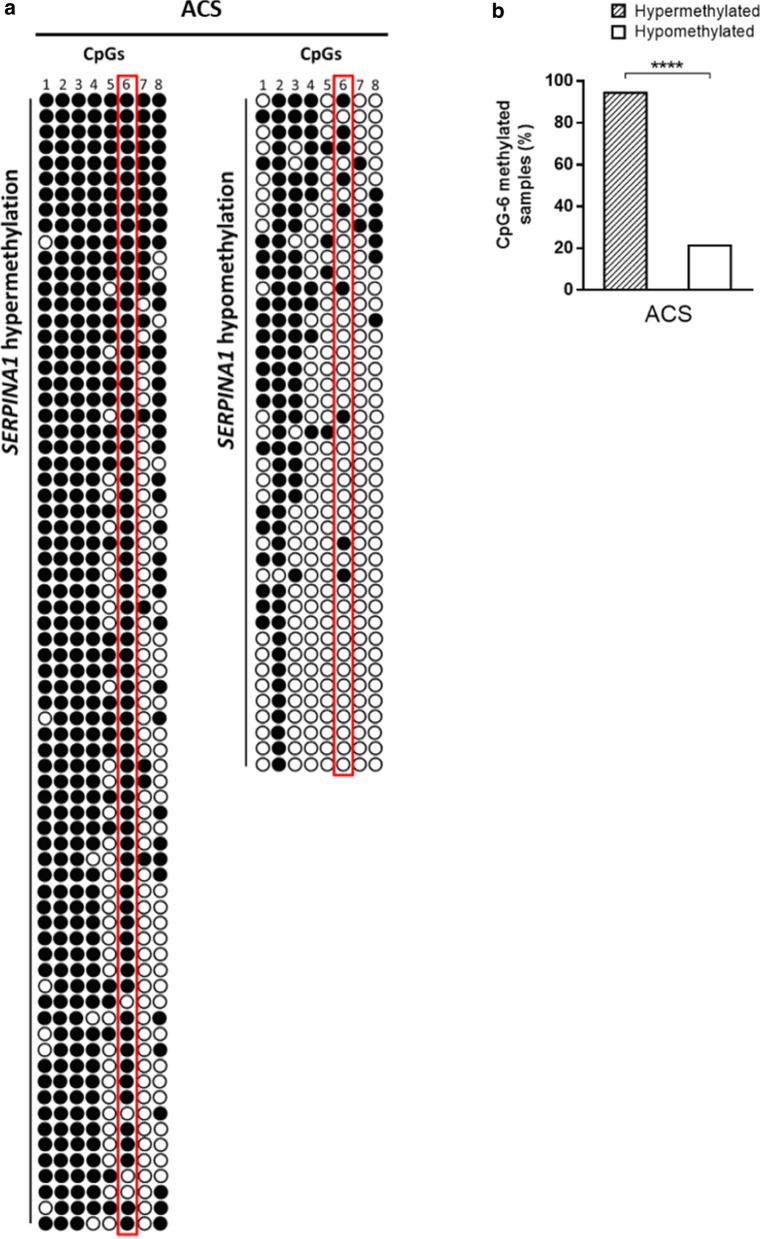
**a** Acute coronary syndrome (ACS) patient’s stratification according to the number of CpGs methylated in *SERPINA1*, i.e., hypermethylation and hypomethylation. Methylated and unmethylated CpG-6 in *SERPINA1* hypermethylated and hypomethylated samples, respectively, are reported. Samples showing more than or equal to 5 out of 8 (≥ 50%) methylated CpG dinucleotides were considered hypermethylated. Filled-in and clear circles represent methylated and unmethylated CpGs, respectively. The CpGs within the *SERPINA1* promoter are numbered across the top of the grid. Each row represents one PCR product/analyzed sequence. Vertical red box highlights CpG-6 dinucleotides across hypermethylated and hypomethylated samples. **b** Frequencies of methylated CpG-6 in *SERPINA1* promoter hypermethylated and hypomethylated ACS samples. ****p < 0.0001

As CpG-6 dinucleotide has been recently associated with the epigenetic regulation of *SERPINA1* gene by promoter methylation in blood cells from healthy individuals [21], we specifically investigated the methylation status of CpG-6 in *SERPINA1* hypermethylated and *SERPINA1* hypomethylated samples. To this aim, ACS patients were first stratified on the basis of the degree of methylation of *SERPINA1*: 5 to 8 (hypermethylated) and 1 to 4 (hypomethylated). Then, the number of CpG-6 methylated and CpG-6 un-methylated was counted in the two groups (Fig. 3a). CpG-6 was found to be methylated in 68/72 (94.4%) hypermethylated ACS samples and in 9/43 (20.9%) hypomethylated ACS samples (*p* < 0.0001; Fig. 3b).

Since hypomethylated CpG-1 and CpG-8 sites have been associated with lower average lung function phenotypes and COPD [26], we investigated the methylation status of these CpG sites in association with COPD. CpG-1 was found methylated in 24/30 (80%) and 60/85 (70.6%) of COPD+ and COPD−, respectively (*p* > 0.05). Furthermore, CpG-8 resulted methylated in 11/30 (36%) of COPD+ and in 34/85 (40%) of COPD− (*p* > 0.05). These results indicated a lack of association between CpG-1 and/or CpG-8 methylation and COPD.

Moreover, as the mean age was significantly higher in COPD+ (*n* = 30, mean age ± SD: 70 ± 7 years old) than in COPD− (*n* = 85, mean age ± SD: 63 ± 9 years old) (*p* < 0.001), age-related changes in *SERPINA1* promoter methylation of ACS patients were assessed. Results indicate a lack of correlation between age and *SERPINA1* promoter methylation in ACS patients (*r* = − 0.071 and *p* > 0.05) as well as between age and *SERPINA1* methylation in both COPD+ (*r* = − 0.073, *p* > 0.05) and COPD− groups (*r* = − 0.166, *p* > 0.05). These results indicate that *SERPINA1* promoter methylation is age independent in ACS patients with and without COPD. Thus, age did not represent a possible cofounder factor for *SERPINA1* methylation analysis, as reported in healthy subjects [21].

In addition, in order to exclude diabetes as a confounding factor, a correlation between diabetes and *SERPINA1* promoter methylation was assessed. The whole pool of ACS patients (*n* = 115) was stratified in patients with (*n* = 28) and without (*n* = 87) diabetes, and *SERPINA1* hypermethylation rates were compared. A total of 67.9% (19/28) and 60.9% (53/87) of ACS patients with and without diabetes, respectively, presented *SERPINA1* hypermethylation (*p* > 0.05). ACS patients were afterward stratified according to diabetes status in COPD+ with (*n* = 9) and without diabetes (*n* = 21) as well as in COPD− with (*n* = 19) and without diabetes (*n* = 66), and *SERPINA1* hypermethylation rates were compared. *SERPINA1* was hypermethylated in 100% (9/9) of COPD+ patients with diabetes and in 71.4% (15/21) of COPD+ patients without diabetes (*p* > 0.05). Moreover, 52.6% (10/19) and 57.6% (38/66) of COPD− patients with and without diabetes, respectively, presented *SERPINA1* hypermethylation (*p* > 0.05). Thus, no differences in *SERPINA1* methylation status were observed when analyzing groups according to diabetes. These results indicate that the presence of diabetes did not suppose an excluding factor in our study.

### Association of lymphocytes count and SERPINA1 hypermethylation in COPD−

**Fig. 4:**
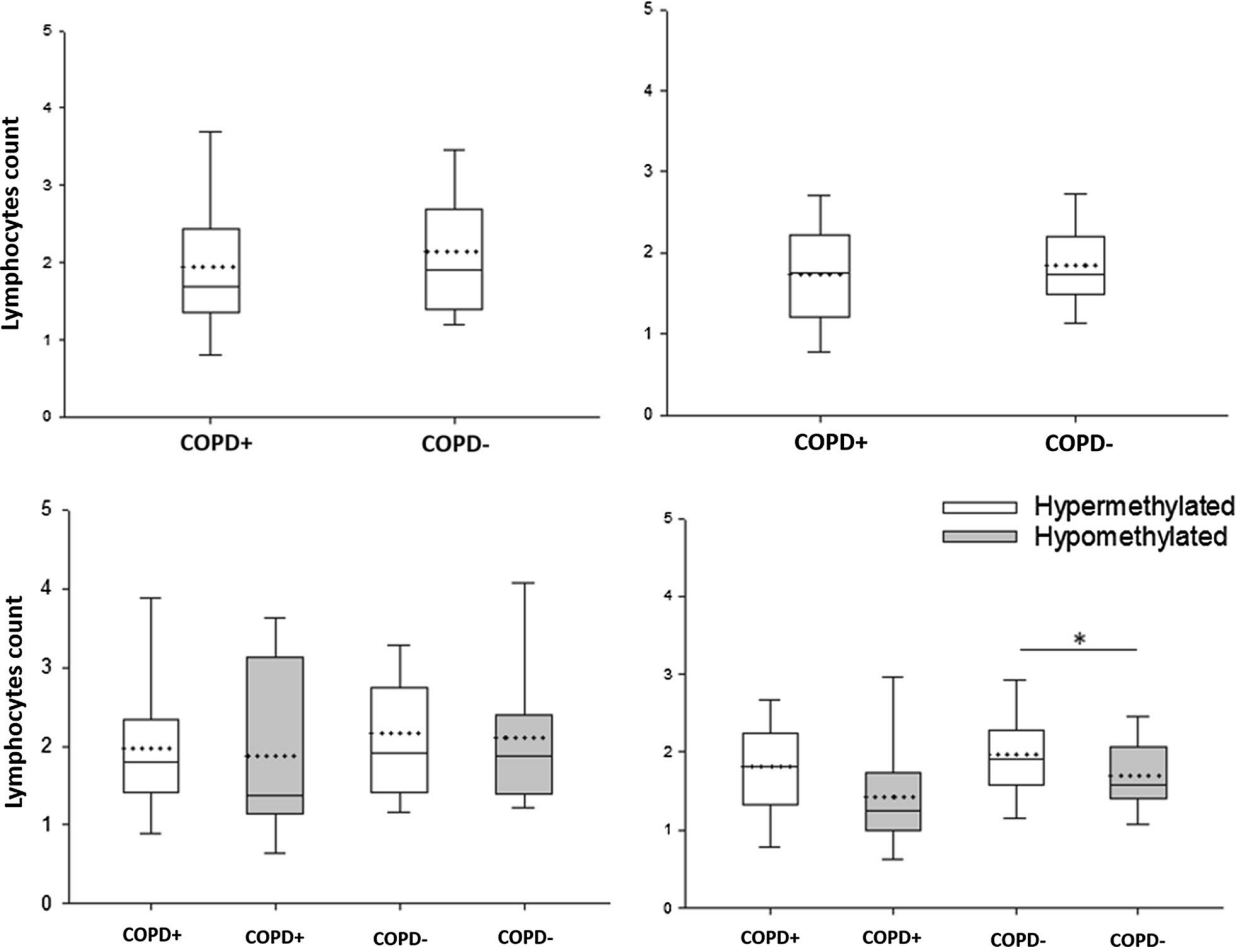
Boxplot graph resuming the lymphocyte count in acute coronary syndrome (ACS) patients with chronic obstructive pulmonary disease (COPD+) and without COPD (COPD−) during patient **a** admission and **b** discharge. Lymphocyte count in COPD+ and COPD− patients in **c** admission and **d** discharge according to *SERPINA1* promoter hypermethylated (white) and hypomethylated (grey) statuses. All panels: the lower (Q1) and upper (Q3) quartile, represent observations outside the 5–95 percentile range. The graph also shows the median (continuous middle line) and the mean (dashed line) of lymphocytes (× 10^3^ cell/µl) per study group. *p < 0.05

High levels of circulating lymphocytes are involved in COPD pathogenesis [36–38]. Therefore, lymphocytes count, available from the hospital database, was analyzed in COPD+ (*n* = 30) and COPD− (*n* = 85) patients at hospital admission and discharge, by multiple linear regression model. Lymphocytes count was 1.955 × 10^3^ ± 0.960 cell/µl in COPD+ and 2.13 × 10^3^ ± 0.98 cell/µl in COPD− at hospital admission (*p* > 0.05; Fig. 4a). At hospital discharge, lymphocytes count was 1.73 × 10^3^ ± 0.73 cell/µl in COPD+ and 1.86 × 10^3^ ± 0.57 cell/µl in COPD− (*p* > 0.05) (Fig. 4b).

Additionally, the relationship between *SERPINA1* promoter methylation and lymphocytes count was investigated in admitted/discharged COPD+ and COPD− patients. At admission, lymphocytes count was similar in COPD+ with *SERPINA1* hypermethylation (1.97 × 10^3^ ± 0.92 cell/µl) and COPD+ with *SERPINA1* hypomethylation (1.88 × 10^3^ ± 1.05 cell/µl) (*p* > 0.05; Fig. 4c), as well as in COPD− with *SERPINA1* hypermethylation (2.16 × 10^3^ ± 0.95 cell/µl) and COPD− with hypomethylation (2.11 × 10^3^ ± 1.01 cell/µl) (*p* > 0.05; Fig. 4c). At discharge, lymphocytes count was similar in COPD+ with *SERPINA1* hypermethylation (1.81 × 10^3^ ± 0.69 cell/µl) and COPD+ with hypomethylation (1.42 × 10^3^ ± 0.73) (*p* > 0.05; Fig. 4d), whereas it was found to be higher in COPD− with *SERPINA1* hypermethylation (1.98 × 10^3^ ± 0.6 cell/µl) than in COPD− with *SERPINA1* hypomethylation (1.7 × 10^3^ ± 0.48 cell/µl) (*p* < 0.05, Fig. 4d).

Considering that the level of neutrophil count and neutrophil/lymphocyte ratio (NLR) is important prognostic predictors to assess the degree of inflammation [39, 40], these parameters were evaluated in ACS patients with and without COPD at both hospital admission and discharge. Neutrophil count was 5.95 × 10^3^ ± 2.06 cell/μl in COPD+ and 6.11 × 10^3^ ± 2.66 cell/μl in COPD− at hospital admission (*p* > 0.05). At hospital discharge, neutrophil count was 4.91 × 10^3^ ± 1.36 cell/μl in COPD+ and 5.56 × 10^3^ ± 1.84 cell/μl in COPD− (*p* > 0.05). NLR resulted to be 3.78 × 10^3^ ± 2.51 cell/ μl in COPD+ and 3.47 × 10^3^ ± 2.17 cell/μl in COPD− at hospital admission (*p* > 0.05), and 3.39 × 10^3^ ± 1.88 cell/ μl in COPD+ and 3.24 × 10^3^ ± 1.44 cell/μl in COPD− at hospital discharge (*p* > 0.05). Furthermore, no differences in both neutrophil count and NLR were found when comparing hospital admission and discharge values, for both COPD+ and COPD− groups (*p* > 0.05). These results indicate that measuring for neutrophil count and/or NLR in ACS patients does not enable to differentiate between COPD+ and COPD− ACS patients and cannot help in predicting which ACS patients are at risk of developing COPD.

## Discussion

In the present study, we investigated the methylation status of *SERPINA1* gene promoter in blood samples from ACS patients with (COPD+) and without (COPD−) COPD. Our results show that *SERPINA1* promoter methylation is higher in COPD+ patients compared to COPD−. Therefore, *SERPINA1* gene promoter hypermethylation may be a potential biomarker for detecting COPD in ACS patients.

*SERPINA1* is constitutively expressed in hepatic cells [6]. *SERPINA1* is also expressed in inducible manner in hepatocytes and blood cells during the course of the inflammation, infections and late pregnancy [16, 17, 19–21]. In PBMCs of healthy individuals the *SERPINA1* promoter is hypermethylated except in women in advanced stage of pregnancy, suggesting the gene silencing under normal physiological conditions and the gene expression induction when circulating AAT is needed to be increased [19–21]. In this study, *SERPINA1* gene promoter was found to be hypermethylated in 62.6% of ACS patients. The prevalence of *SERPINA1* hypermethylation was higher in COPD+ (80%) than in COPD− (56.5%) patients (*p* < 0.05). This result suggests an association between *SERPINA1* hypermethylation and COPD+ leading to the hypothesis that lack of AAT expression in blood cells may play a role in the development of COPD in ACS patients. The relevance of AAT in COPD is well known in smokers carrying mutations in *SERPINA1* gene where protein deficiency synergizes with smoking in exerting strong adverse effects on lung function, causing COPD [11]. As ACS patients enrolled in this study are all smokers, the adverse effects on lung functions may be mediated by altering promoter methylation in *SERPINA1*.

In addition, we specifically analyzed methylation status at CpG-6 site. The dinucleotide CpG-6 falls within a CCGCCC box regulatory consensus region of *SERPINA1* promoter. The CCGCCC box is located 46 nucleotides upstream of the transcription start site in *SERPINA1* promoter, and it is recognized by transcription factors (TFs) to recruit RNA polymerase, thereby inducing transcription [41]. Methylation at CpG-6 could prevent CG-box-TFs interaction, thereby inducing gene transcriptional repression [42]. In our previous study, we have found that methylation status of CpG-6 reflects the methylation status of *SERPINA1*, being methylated in *SERPINA1* hypermethylated samples and unmethylated in *SERPINA1* hypomethylated samples [21]. In this study, nearly 95% of *SERPINA1* hypermethylated samples had CpG-6 methylated, and 79% *SERPINA1* hypomethylated samples had CpG-6 unmethylated. These results extend our previous observations and confirm that CpG-6 may play a pivotal role in epigenetic regulation of *SERPINA1* in blood cells [21]. Therefore, the methylation status of CpG-6 may represent a useful marker of expression of *SERPINA1* in blood cells.

As high levels of circulating lymphocytes play a role in COPD pathogenesis [36–38], lymphocytes count was analyzed in COPD+ and COPD− patients at hospital admission and discharge. Lymphocytes levels were similar in COPD+ and COPD− patients, both at hospital admission and discharge. Nevertheless, COPD− patients carrying *SERPINA1* hypermethylated had circulating lymphocytes higher than COPD− patients carrying *SERPINA1* hypomethylated, at hospital discharge (*p* < 0.05). This result suggests that COPD− patients carrying *SERPINA1* hypermethylated may counteract inflammation less efficiently than COPD− patients carrying *SERPINA1* hypomethylated. Therefore, we hypothesize that *SERPINA1* hypermethylation may represent a risk factor for developing COPD in ACS patients at risk (smokers) with high levels of circulating lymphocytes in the presence of pharmacological treatments following percutaneous coronary intervention. In this view, ACS patients at risk of developing COPD might be identified by combining epigenetic and hematologic parameters. Follow-up studies should be carried out to verify whether hypermethylated *SERPINA1* and lymphocyte count may be useful markers to predict the development of COPD in COPD− patients.

Few studies have explored *SERPINA1* methylation in COPD pathogenesis. Our findings are partially in line with a recent meta-analysis study of tobacco-smoke exposed children and adult smokers, which reported association between methylated CpGs located about 32 kb downstream of *SERPINA1* gene and decline in lung functions [26, 43]. Our data are discordant compared to a previous family-based study of smoking subjects with and without a history of COPD, which found two hypomethylated CpG sites, the CpG-1 and CpG-8 of this study, associated with lower average lung function phenotypes and COPD [26]. It should be pointed out that comparisons between our data and those reported above are difficult to be made, as both patient enrolment criteria and methodological approaches used herein are different. Indeed, we enrolled ACS patients, who were stratified according to the presence/absence of COPD, while previous data were obtained from predominantly smoking adults with/without COPD [26]. About methods, unlike previous investigations that studied epigenome-wide associations, our study was specifically designed to assess the methylation profile of *SERPINA1* gene promoter in blood cells from ACS patients.

Our study presents some limitations. First, we studied the epigenetic regulation of *SERPINA1* through promoter methylation without validation analyses, such as AAT mRNA and protein expression. However, in our previous study conducted on healthy subjects we determined an association between variations in *SERPINA1* promoter methylation in PBMCs and changes in AAT circulating levels [21], while promoter methylation of *SERPINA1* in association with gene expression inhibition has been demonstrated in animal models [44–46]. Second, we did not analyze mutations in *SERPINA1* gene. Since about 1% of individuals carry mutations in *SERPINA1*, it is possible that some COPD+ may be associated with mutations in this gene. Considering that most of the gene mutations occur in the coding region [47], we may infer that SERPINA1 gene regulation by methylation in blood cells is not affected.

In conclusion, the present study shows, for the first time, that SERPINA1 gene promoter is hypermethylated in blood cells from COPD+ patients, compared to COPD−. Further, we show that CpG-6 methylation status reflects the methylation status of *SERPINA1* promoter. We also found that COPD− patients with *SERPINA1* hypermethylated present higher lymphocytes levels, in the presence of pharmacological treatments following percutaneous coronary intervention, than COPD− patients with *SERPINA1* hypomethylated. Collectively, our data indicate that *SERPINA1* hypermethylation may play a role in the development of COPD in ACS patients. Therefore, *SERPINA1* promoter methylation may be a potential biomarker for detecting/predicting COPD in ACS patients.

## Data Availability

All data generated or analyzed during this study are included in this published article.

## Abbreviations

COPD: Chronic obstructive pulmonary disease
ACS: Acute coronary syndrome
AAT: For alpha1-antitrypsin
CpG-6: CpG number 6
TFs: Transcription factors
